# Protection of gender health and fight against gender violence during the COVID-19 pandemic: the experience of our street clinic in a disadvantaged suburb of Rome Metropolitan City

**DOI:** 10.1186/s12905-023-02595-7

**Published:** 2023-08-16

**Authors:** Suleika Urbano, Elisabetta Gobbi, Valeria Florio, Aurelia Rughetti, Lucia Ercoli

**Affiliations:** 1 Istituto Di Medicina Solidale Onlus, Rome, Italy; 2 Gynecology Department, Fatebenefratelli Hospital, Rome, Italy; 3 Department of Experimental Medicine, Sapienza University of Rome, Rome, Italy; 4 Department of Biomedicine and Prevention, Tor Vergata University, Rome, Italy

**Keywords:** Health inequalities, Intimate partner violence, Poverty, Screening, Fragile population, COVID-19, Women health

## Abstract

**Object:**

In this study, we evaluated health, social inequalities and risk to gender violence of women living in a disadvantaged degraded suburb of Rome Metropolitan City, during COVID-19 pandemic.

**Methods:**

The study included 779 women referring to primary care services of Medicina Solidale Institute for gynecological/breast examinations (209), medical and support aid for the children (383) and COVID-19 test execution (187).

**Results:**

The data show that most women (68%) were unemployed or had an irregular job. The request of support varied depending on the ethnicity: while healthcare support was requested mostly by African female community, the COVID-19 test, mandatory for public transportation and work, was a need of the east-european community. Both these communities referred to Medical Solidale primary care service for the healthcare and food/clothing support for their children.

It is interesting to note that the requests from the Italian women community was elevated in terms of personal healthcare, support for the children and COVID-19 test execution.

The access to the national health system (NHS) resulted a complex administrative procedure despite the original social-ethnic communities. The vast majority of women lacked awareness of their crucial role for supporting the family entity, while inadequacy was commonly reported.

**Conclusions:**

This study confirms a critical condition for women living in disadvantaged neighborhoods, whose vulnerability is further worsened by the limited access to primary care assistance with serious consequences for health and quality of life. Prevention and treatment, especially for the most vulnerable subjects, should be a priority for the public health system.

## Introduction

Coronavirus Disease 19 (COVID-19) has seriously affected all tiers of the population, with an even worse impact on the most vulnerable persons. Indeed, healthcare, educational, social services networks have been dramatically compromised by the pandemic. Furthermore, the containment measures adopted during the pandemic sprout as well as in the following periods have heavily hit those in most vulnerable circumstances and dramatically worsened disparities for healthcare, as well as primary life services with huge economic impact [1, 2]. There is a compelling urgency to globally and nationally identify those point of weakness and strengths of the health, economic and social systems to be better prepared for next pandemic/health crisis and improve equity, social justice, solidarity [3, 4].

Italy was the first European country to face the COVID-19 pandemic, and radical containment measures were adopted in the first phase of pandemic (March 2020), but also in the following year. During the second lockdown (April 2021), access to public transport was conditioned by the possession of the reinforced green pass and for not vaccinated people was therefore not possible to travel, limiting access to health centers as well, also to undergo vaccination [5–8]. These restrictions were maintained until the 31^st^ of April 2022.

The lack of vaccination certificate led to the loss of work especially for women employed as caregivers and domestic workers, significantly worsening the already precarious economic conditions of vulnerable populations.

The Medicina Solidale Institute primary care services (https://medicinasolidale.org/), located in disadvantaged suburbs of Rome, aim at protecting women's health with dedicated general and specialist visits for them and their children, offering gynecological, obstetrical and breast examinations, ultrasound scans and psychological interviews performed free of charge.

During the pandemic, the women who referred to our centers have yet highlighted precarious situations, economic and social problems, difficulties in using the national health services for reasons related to the language, the lack of knowledge of the system and the lack of adequate Documentation [9–11].

In this retrospective study we describe the experience of the Medicina Solidale Institute primary care centers and street units in treating vulnerable women and their children during the COVID-19 pandemic in disadvantaged suburbs in Rome.

## Materials and method

### Medicina Solidale Institute primary care center and patient population

The Medicina Solidale Institute primary care center is located at the east/south east boundaries of the Rome metropolitan city, just outside the major highway around Rome (https://medicinasolidale.org/). Medical, psychologic and primary social care supports are offered to families (mainly women and children).

Between May 2020 and March 2022, a total of 779 women had access to the Medicina Solidale Institute primary care center. As defined by the Steering Committee of the Medicina Solidale Institute, informed consent was prepared in accordance with the ethical principles for medical research involving human subjects following the Declarations of Helsinki and in accordance with the Italian national guidelines for privacy protection in healthcare. The Steering Committee further established that informed consent should have had administered for each access to the care services of Medicina Solidale Institute. The health care center of Medicina Solidale Institute was open from 8,30 to 16, from Monday to Friday, assuring several health, social and educational services. Following specific time-table. The services described herein were available trice/weekly (Mo, Wed, Fri, 10–15). Access to the services of Medicina Solidale Institute was free of charge and independently by ethnicity and social status. It occurred mainly by word of mouth among the people coming from the neighborhood as well as from other distant area of Rome metropolitan city, mainly by public transport.

Upon acceptance of informed consent, demographic and socio-economic variables were collected for each woman, i.e. date of birth, country of origin, religion, level of education, date of arrival in Italy, date of first access to the service; marital status; children; accommodation types; fixed residence or not; profession; personal document; general practitioner.

The women who accessed for personal health care were welcomed by a team composed of a doctor, a gynecologist, a midwife, a social and cultural mediator, and a psychologist [9].

Further information regarding past and proximate clinical history were collected. Past clinical history variables consisted in: brief family history (with particular reference to family history of breast, uterus and ovary neoplasms); physiological history (in particular information regarding the age of the menarche, any menopause, the trend of menstrual cycles, the start date of the last menstrual cycle, obstetric history); past medical history (with attention to gynecological-obstetric pathologies and any sexually transmitted disease (STDs)); alcohol/smoking; obstetric history of previous pregnancies (parity, type of birth, voluntary termination of pregnancy, desired or not pregnancy, use of contraceptive methods) and gestational outcome (type of birth, date of birth of the newborn, weeks before delivery, execution of episiotomy, weight of the newborn at birth, Apgar index, breastfeeding, structure chosen by the woman for delivery, post-partum checks, important figure at the time of delivery).

Also, proximate clinical history variables were collected in particular: reason for the visit; gynecological physical examination; recommended blood and/or instrumental tests; any prescribed therapies.

In the case of women arrived for pregnancy, data relating to the current pregnancy were also collected (weeks at the time of the first visit, checks carried out during pregnancy, virological and infectious tests carried out, ultrasound scans carried out, swabs carried out, pap tests, course of pregnancy, birth accompaniment course, ECG, presumed date of childbirth).

In addition to the gynecological physical examination, all patients were offered screening for the neoplastic pathology of the uterine cervix, including the execution of the Pap test (unless the patient had documentation of the execution of this during the previous year).

The women undergoing gynecological visits were also offered to have a semi-structured interview for personal support. In this case, a one-to-one interview with the team psychologist was carried out, inspired by the reason of the visit, the emotional status of the woman and/or the demographic, socio-economic and medical variables already collected. The semi-structured interview was conducted following a defined series of questions to investigate the socio-cultural and emotional network of the woman. Topics of the interview was related to the personal living and nutrition habits; organization and planning of the family life; children education (possible support and aid, communication with the school-team); presence of elder children in other countries. The relationship with the partner was also a point of discussion. The woman was offered to return for a second interview on a voluntary basis.

### COVID 19 test

To detect COVID-19 positivity, a SARS CoV2 Antigen Rapid Test (by nasopharyngeal swab) (Hangzhou AllTest Biotech Co, China) was performed following manufacturer’s instructions. The test report was provided to each patient. Upon positivity detection, indication of the correct isolation procedure and medical suggestions were provided.

## Results and discussion

### Distinct needs profile of women with social-economic frailties during pandemic period

During the period between May 2020 until March 2022, distinct COVID-19 waves occurred and access to public transport as well as work activities was conditioned by the possession of the reinforced green pass. Travelling on public transport was not permitted for not vaccinated people, therefore limiting access to health centers as well.

Furthermore, the sudden changes in the anti-COVID 19 legislation adopted in Italy during this period have increased complexity of healthcare and social style habits, making it more difficult for people living in degraded suburbs.

**Fig. 1:**
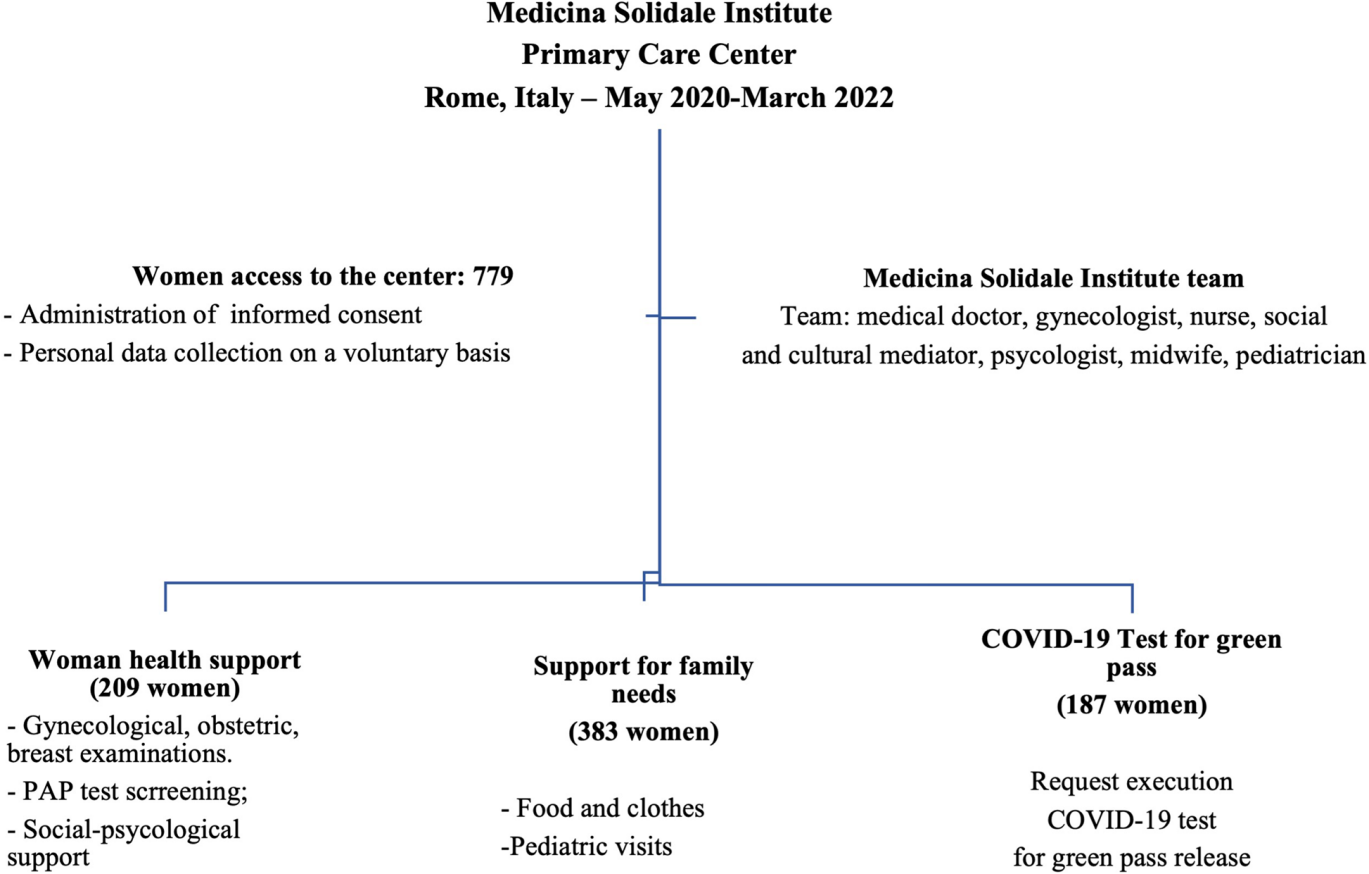
Schematic diagram representing the main features of the primary care services of Medicina Solidale Institute for women during the period May 2020—March 2022

During the period between May 2020 until March 2022, a total of 779 women benefited of the healthcare services provided by the Medicina Solidale Institute health care services. Among these, 209 women requested (27%) gynecological examinations and assistance, 383 ones (49%) accompanied their children for medical control and/or to get food and clothing, while 187 women (24%) requested assistance for the COVID-19 tests (Fig. 1). The information presented herein were collected at the first access of each woman and numerosity of the above-mentioned groups was defined by the real user requests. The data were retrospectively analyzed.

### Frail women and request for gynecological assistance and support

In the considered period (May 2020-March 2022), 209 women had newly access to the street clinic service for gynecological support with a median age range of 35.8 years (16–70). The first medical access was due to gynecological examinations (148), obstetric visits for pregnancy (43) and breast examinations (36). A total of 459 visits were carried out during the period (2.2 average visits for each woman).

**Fig. 2:**
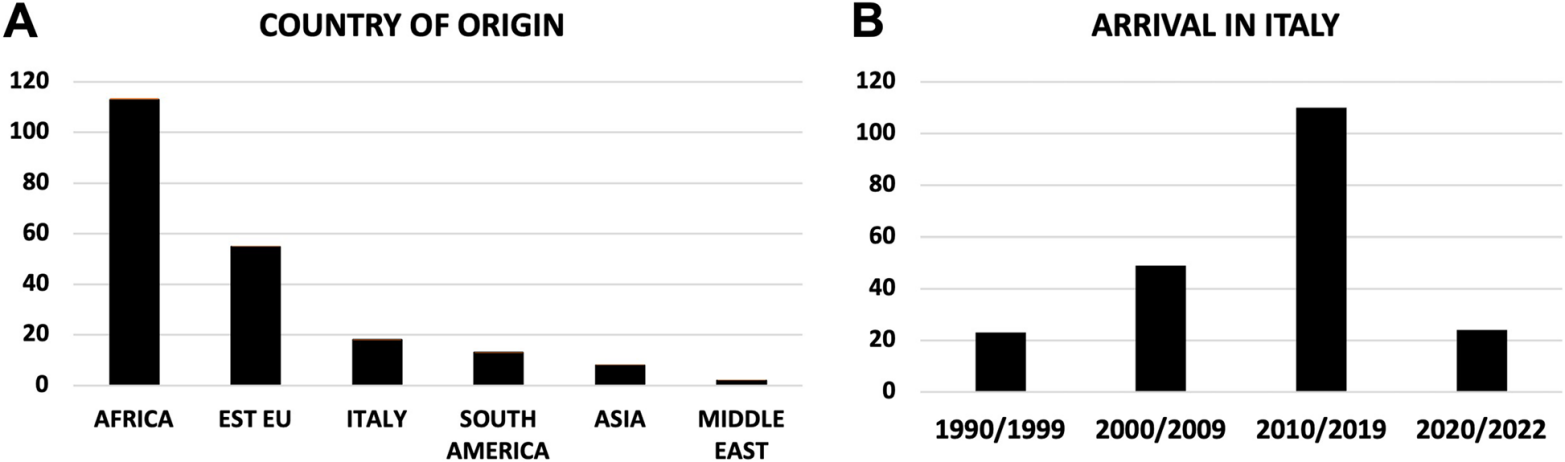
Socio-demographic data of the women acceding to the street clinic units for health care needs (209). Histograms represents subject distribution on the basis of the country origin (**A**) and period of arrival in Italy (**B**)

Most of the women were from Africa (mainly Nigeria and Marocco) and East Europe (mainly Romania) (Fig. 2A).

Interestingly, the third group by number density was made by Italians thus suggesting that several Italian women did not use the public Italian National Health Service (NHS). Most of the women has been in Italy since before the pandemic period: 53% arrived between the 2010 and 2019, while only 12% arrived during the pandemic period (Fig. 2B). At the interview, 74% of the women resulted to have a stable relationship (56% were married and 18% had a partner) and 78% were mothers (of at least one child (19%) without an extended family support network (Table 1).

**Table 1:** Social-demographic and health characteristics of the women requesting gynecological assistance and support

	**Nr of women (total 209)**	**%**
**Reason first visit**		
*Gynecological check-up*	130	62,2
*Breast examination*	18	8,6
*Gynecological and breast check up*	18	8,6
*Pregnancy*	43	20,6
**Marital status**		
*Married*	116	55,5
*Maiden*	46	22,0
*Cohabitant*	37	17,7
*Devorced*	7	3,3
*Widow*	3	1,4
**Parentality**		
*No child*	46	22,0
*1 child*	40	19,1
*1* < *child*	123	58,9
**Employment**		
*Unemployed*	141	67,5
*Housewife*	31	14,8
*Housekeeper*	14	6,8
*Caregiver*	6	2,8
*Hairdresser*	5	2,4
*Student*	5	2,4
*Other*	7	3,3
**NHS Document**		
*CF*	152	72,7
*STP*	11	5,3
*ENI*	6	2,9
*No valid document*	40	19,1
**General practitioner**		
*No*	93	44,5
*Yes*	116	55,5

Type of social cards for access to public health services: *CF* Tax Code, *STP* Temporarly present foreigner code, *ENI* European non resident citizen code

The majority of them (67,5%) were unemployed at the time of the access; those who had a job were mainly housekeepers, hairdressers, and caregivers (Table 1). This data was expected and certainly conditioned by the emergency relating to the COVID-19 spread. Indeed, the already few women who had managed to find a job despite the difficulties before the pandemic were fired or forced to stay at home both for work-related issues and for family management issues the first COVID 19 lockdown.

It is interesting to note that only 10% recognized their role as housewife, although 74% admitted to have a stable relationship. This suggests that the women did not consider their fundamental role as care-giver for the family.

Among the women who acceded for the first time our primary care services, 25% (43) were pregnant and required the midwife support, while 75% requested gynecological or breast examination or both (130, 18 and 18, respectively) (Table 1). It is interesting to note that the 19,1% of the women who requested health support did not have any valid document to get access to the NH care system, while the 81,9% did have the possibility to get access to public health service.

Access to the Italian NHS can occur in the presence of the following documents: tax code, European citizen not registered (ENI) and Temporarily present non-Italian and EU citizen (STP) (Table 2).

**Table 2:** Documents and access to the NHS

	**Document**	**Authorizing institution**	**Requirements**
C.F	TAX CODE	Revenue Agency	Italian citizens or Foreigns who certified work. Required Italian official residency/domicile
STP	STP code	ASL (Local Health Agency)	Non EU citiziens, not regularly registered. Indigence declaration. Six month validity
ENI	ENI code	ASL (Local Health Agency)	European citiziens without Italian residence and no health care assistance from the origin country. Six month validity

Mandatory for obtaining any of those is to demonstrate a regular residence plus a regular work contract for foreigners. In addition, ENI or STP cards are renewed on 6-month period, and this administrative work-flow makes really hard to have an active NHS card and get access to public health care services on a regular basis.

Indeed, despite the 20,1% of the women did not have the NHS card, more than 40% of the women declared to not have a general practitioner (Table 1), because they no longer had a residence, or the tax code had expired and they could not renew it due to the unemployment condition. Although most of the women arrived in Italy before 2015, their social and living conditions did not permit them to set-up a regular living style and enabled them to have guaranteed the health service. It is interesting to note that religious origin is equally distributed between different religious confessions and monotheisms, indicating that in the outpatient clinic women did not encounter any cultural barriers.

Among the 148 women who requested the gynecological support, 61% had not performed a pap-test screening before. This is a rather relevant figure, considering the average age of women and the role of pap test as the cornerstone of cervical cancer prevention programs. This may be due to distinct reasons: a scarce consciousness of the own health protection and the impaired access to the public health system.

**Fig. 3:**
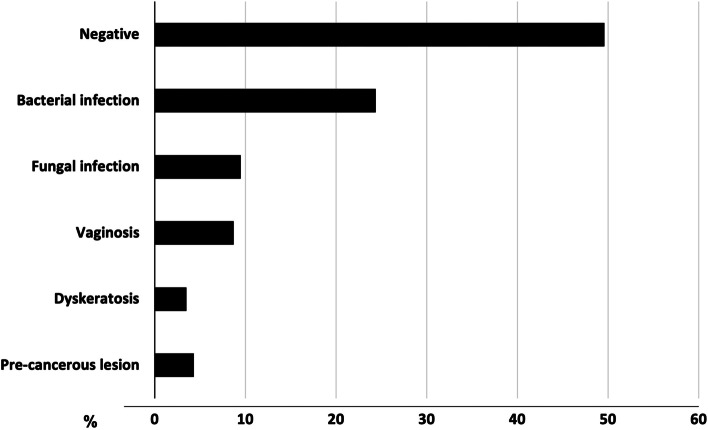
PAP-Test Screening and diagnosis of gynecological infection/morbidity. Pap-test screening was accepted by 110 women out the 148 undergoing gynecological examination

The PAP test screening was accepted by 115 women out of 148 who requested gynecological support (77%), thus indicating that the primary health care intervention was able to raise awareness for self-health care in a large proportion of the women. The PAP test results indicated that 57 patients (49,5%) resulted negative to any morbidity, while the remaining showed the presence of pathological states due to bacterial or fungal infection (24,4% and 9,5%, respectively), vaginosis (8,7%) and dyskeratosis and pre-cancerous lesion, 3,5% and 4,3%, respectively) (Fig. 3).

Another important point is related to the 41 pregnant women who requested health care during their pregnancy. All of them arrived to term before the ending of the study.

Despite the different life stories, a common trait has been the request for further information and help to decide to be vaccinated or not. The acceptance of anti-COVID-19 vaccination has been a critical issue and several social as well as personal factors might have influenced it [12, 13]. Medical advice combined to higher education were crucial factors to guarantee adherence to vaccination schedule.

### Survey of institutional violence and social, economic and health marginalization of frail women at the clinic street center

At the same time as the obstetrician-gynecological visits, personal interviews were held in order to detect risk indicators of institutional violence and social, economic and health marginalization [14].

Before the obstetric-gynecological visit, all 209 women, newly accepted in the clinic services, underwent an interview at the first visit that included a collection of personal and social data.

The interview was developed to assess environmental risk conditions such as poverty, number of off-spring, poor family support, lack of a good social network, presence of stressful events close to childbirth, neglect, absence of a partner or presence of an inadequate or even abusive partner [15–18].

The elements that most significantly emerged from the interviews and which have been aggravated by the emergency situation relating to the spread of COVID-19 concerned the lack of work (almost total for women and partial for their companions who very often did not have one), regular or adequate salary to contribute to the sustenance of the entire family, and the absence of a supportive social network.

Most of the interviewed women were unemployed and the few who carried out cleaning services in hotels or homes were fired as consequence of the COVID-19 pandemic. This condition of social and economic vulnerability exasperated an already fragile condition: most of their partners did not have a job or had lost it due to the pandemic.

For the women living in occupied buildings or camps in the Rome suburbs, the condition was even worse since access to any type of work had already been precluded by the instability and discomfort of the housing conditions which, even just because of their isolated location, had increased physical and psychological distance and/or exclusion from the social and working realities of the area [19, 20].

Many women also report that on-line learning activities, quarantines and the presence of children at home even in the morning, had further precluded the possibility to look for and replace a job.

The end of the pandemic and the complete return to school of their children were identified as the optimal times to dedicate efforts to look for a job.

**Fig. 4:**
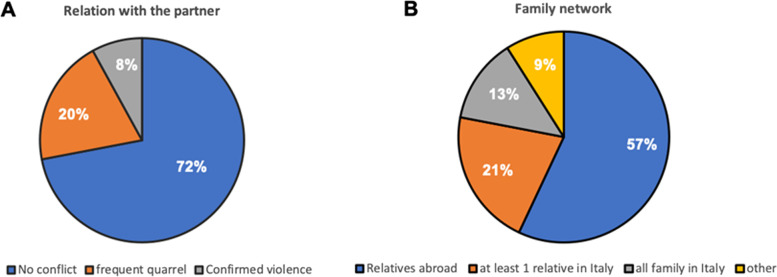
Distress due to conflict with the partner and lack of social and familiar network. Pie charts show the conflict with the partner (**A**) and family network (**B**). The percentages are referred to the total number of the women requesting gynecological/obstetric/breast examination (209)

Despite the suffering of social, family and economic difficulties, most of the women (72%) denied any conflict with the partner, using the phrase "It is all right" seldom accompanied by adequate arguments to support this position; 20% of the women interviewed admitted frequent quarrels with their partners. Only 8% confirmed that somehow suffered violence (Fig. 4A).

Women who claimed to have a conflict with their partner reported that they argue mainly for economic reasons. Indeed, Intimate partner violence (IPV) has been considered a pandemic within the COVID-19 pandemic [19, 21]. Indeed, the effects of lockdowns, quarantine combined to the isolation at home with abusive partners, as well as economic worries/loss of a job could significantly facilitate violence against women.

This dreadful and dangerous combination of events at the same time have diminished women's chances to seek for help, with a strong negative impact on their life [22].

We believe it is mandatory, when possible, to deepen this particular aspect, to understand the dynamics of conflicts and possible hidden aspects of abuses (not necessarily physical) that the same woman does not recognize, to be able to perform an optimal support, even if sporadic [23–25].

The psychological support was offered to each interviewed woman, however only 9 of them came back for subsequent interviews: this data indicates how the psychological distress is not considered as an aspect to care of.

Another relevant fact that describes the condition of vulnerability and marginalization of the women involved is the lack of a relational support network that is fundamental, furthermore in times of difficulties, also related to pregnancy and the first years of children's life [26].

Again, the data describe a rather negative situation. Fifty-seven% of women did not have relatives in Italy and only 21% had a family member residing in Italy (Fig. 4B). Furthermore, women who declared that they had their whole family in Italy could not always take advantage of the resulting benefits since they often were women who lived in fields where the enormous social hardship was certainly not mitigated by the presence of the family. In the future, the presence of a network of friends could be investigated more thoroughly, which, as an alternative to the family, could equally provide the necessary support in some moments of need such as pregnancy or post-partum [27].

The absence of a relational network, economic stability, the difficulty of accessing care are not the only factors that weigh on the health and freedom of these women; the difficulty of schooling of the children has also been added. Already in 2019 it was noted that 26/100 women had unschooled children, especially in the first 6 years of life. This has worsened, due to the COVID-19 pandemic restriction as many children have not been able to adopt Distance Learning properly and skipped school days that were precious for their healthy development. The non-schooling of the children obliged the mothers to stay at home and had not allow them to carry out work activities [28]. During the interviews, it was found that the women who were first admitted to our primary care centers in 2021 were not aware of the existence of screening programs for the protection of female health and were unaware of the meaning of the PAP test and other investigations, especially those relating to the early diagnosis of sexually transmitted infections. During the interviews it was possible to deepen the importance and function of surveillance programs relating to reproductive health and the protection of pregnancy, and the methods of carrying out some tests, bringing new knowledge and greater awareness of health protection.

### The street clinic center as a resource for women support in managing COVID-19 derived social constrains

During the period between May 2020 and March 2022, we have also observed that a large group of women had access to the street clinic center to ask for support for the basic every-day life activities and taking care of the children and COVID19 test for obtaining the green-pass for public transportation (570 women, 73%).

In particular, 383 mothers (49% of the total access) accompanied their children to the street clinic for a total of 605 pediatric visits; however, only 115 mothers (30%) were available to reveal personal information and very few of them participated to the women dedicated medical screening.

**Fig. 5:**
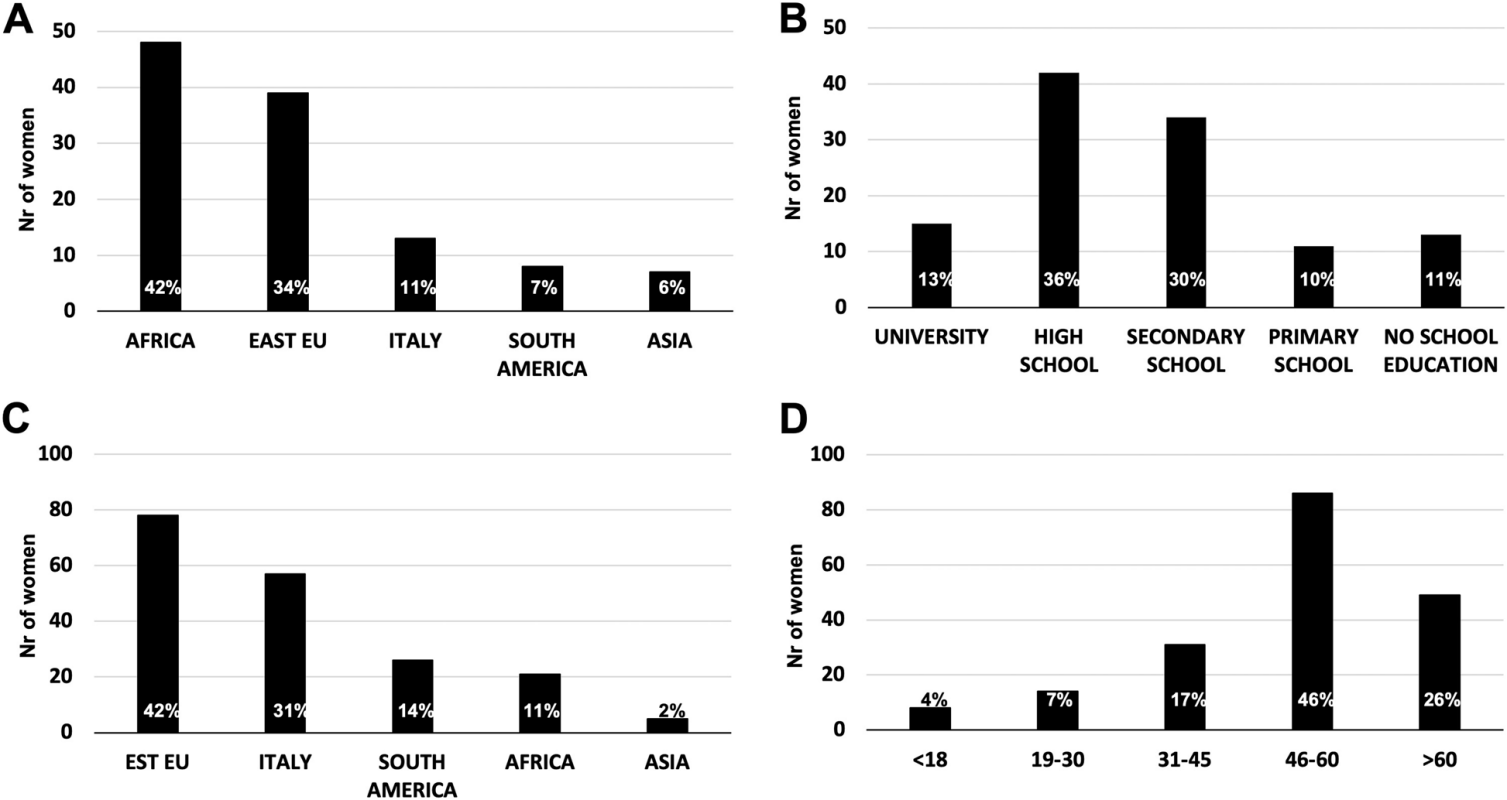
Socio-demographic data of the women acceding to the primary health care center of Medicina Solidale Institute for family/children support (**A**, **B**) or COVID-19 test (**C**, **D**). Data regarding the women asking for family/children support were reordered for 115 women out 383: **A**) country of origin, **B**) Educational status level. Data regarding the women asking for COVID-19 test refer to the 187 women: **C**) country of origin, **B**) Age. Histograms represent the number of women and percentage respect to the groups analyzed are reported within each bar

In this group, African women were the most present, followed by Eastern European and Italian mothers (Fig. 5A). It is interesting to note that the 80% of the mothers who asked for sustaining the children and supporting the family (food, clothes etc.), had medium–high education (Fig. 5B).

This is indirect evidence that also families with medium–high education levels have been in the condition to use the street clinic point care, while not accessing to the NHS, and it strongly suggests that the street clinic unit has become landmark for the social support. Furthermore, the results her presented showed that women had reinforced their role as care-giver for the family to detriment to their own health-care.

Another request from women coming to the Primary health care center of Medicina Solidale Institute was the execution of COVID-19 test. Indeed, the requirement of green-pass had pushed a large group of women (187, 24% of the total access) to ask for anti-COVID-19 tests that were performed for free.

It is interesting to note that in this case, the prevalence of women was from Eastern Europe (42%) and Italy (31%), thus suggesting that the COVID-19 test was required for transportation or work (Fig. 5C). This hypothesis is also reinforced by the fact that the test was preferentially requested by women with age comprised between 30 and 60 years, that corresponds to the working age range (Fig. 5D). This study presents some limitations: the number of the cohort, although significant for the suburbs area of Rome in which Medicina Solidale institute is based, is small to reach a statistical significance compared to the whole Rome metropolitan city. Some women (63) have been not included in the study because of lacking information. The personnel routinely operating at the clinical health care center is on voluntary basis, and COVID-19 had also impacted their availability. Furthermore, the COVID-19 pandemic had a strong impact on lowering the private funding for the Medicina Solidale Institute, therefore availability of reagents could be reduced during the first phases of the pandemic, while support for food and clothes, provided by private citizens always was available. These events had impact at same extent the efficacy of data collection. Despite the limitations above described, this study provides an invaluable insight of the health and social condition of frail women in disadvantages area of Rome capital.

## Conclusions

The COVID-19 pandemic proved to be a health determining factor in an increased inequality in accessing to health services, impairing working conditions, altering familiar balance and exacerbating social isolation of frail population. Women have played a crucial role in balancing the distress, absorbing and managing the very stressful experience [29].

Street clinics with low access thresholds and assisted by street units and social mediators appear to be the most suitable intervention strategy to facilitate access to care for socially fragile people and guarantee a social and health care support, otherwise not available. Furthermore, the active offer of oncological screening and health education courses for cancer prevention appear to be the most effective intervention strategies to combat the onset of neoplastic pathology in socially disadvantaged women [30–32].

The other important issue is that the women during this period have taken care of their children asking for medical intervention, without asking for themselves.

The presence of psychologists within the team also ensured a better assessment of the risk of gender violence and accompanying women in a path of awareness of their rights as persons. The experience of Medicina Solidale Institute resembles the real-world experience of the USA safety-net health care system. These structures are health centers that provide a lifeline for underinsured and uninsured people. The COVID-19 pandemic has shown that this system has been critically important in the managing of the emergency and in the implementation of health care during pandemic for the vulnerable communities [33].

These experiences have also shown that safety-net hospitals were more prepared than other providers to support public health departments, and able to reach and support those people that the public health services could not efficaciously serve.

We believe that the action of Medicina Solidale Institute, together with other non-profit private health care services present in Rome, may strongly contribute to sustain the health care and social network for inclusion of the frail and disadvantaged people in Rome.

## Data Availability

The datasets used and/or analyzed during the current study available from the corresponding author on reasonable request.
